# 

**Published:** 1875-05

**Authors:** 


### Books and Pamphlets Received.

Sex in Industry: A Plea for the Working-Girl. By Azel Ames, Jr., M. D. Boston: Jas. R. Osgood & Co., 1875. Buffalo: Martin Taylor.

What Young People Should Know. The Reproductive Function in Man and the Lower Animals. By Burt G. Wilder, M. D. Boston: Estes & Lauriat, 1875. Buffalo: H. H. Otis.

A series of American Clinical Lectures. Edited by E. C. Seguin, M. D., Vol. I, No. IV. Rest in the Treatment of Nervous Disease. By S. Wier Mitchell, M. D. New York: G. P. Putnam's Sons. Buffalo: Martin Taylor.

Annual Report of the Buffalo General Hospital for the year 1874.

An Address on the Climatology of Florida. By A. S. Baldwin, M. D. The President's Address before the Medical Association of the State of Florida.

Annual Report of the Managers of the State Lunatic Asylum, Utica, New York, for 1874.

Ninty-Second Annual Catalogue of the Medical School of Harvard University for 1874-75.

THE BUFFALO

**Medical and Surgical Journal**

PUBLISHED MONTHLY,

**Containing Original Articles, Reports of Medical Societies and Hospitals, Editorial, Reviews, Correspondence, Army News, etc., etc.; including the usual variety of Medical Periodical Publications. Specimen copies sent on application.**

Address Buffalo Medical & Surgical Journal, Buffalo, N. Y.

TERMS OF SUBSCRIPTION, - - $3.00 PER YEAR, IN ADVANCE.

